# Potential of Polyphenols for Improving Sleep: A Preliminary Results from Review of Human Clinical Trials and Mechanistic Insights

**DOI:** 10.3390/nu15051257

**Published:** 2023-03-02

**Authors:** Masanobu Hibi

**Affiliations:** Biological Science Research Laboratories, Kao Corporation, 2-1-3 Bunka, Sumida, Tokyo 131-8501, Japan; hibi.masanobu@kao.com; Tel.: +81-3-5630-7476

**Keywords:** catechins, chlorogenic acids, circadian rhythm, phenylpropanoid, resveratrol

## Abstract

Global epidemiologic evidence supports an interrelationship between sleep disorders and fruits and vegetable ingestion. Polyphenols, a broad group of plant substances, are associated with several biologic processes, including oxidative stress and signaling pathways that regulate the expression of genes promoting an anti-inflammatory environment. Understanding whether and how polyphenol intake is related to sleep may provide avenues to improve sleep and contribute to delaying or preventing the development of chronic disease. This review aims to assess the public health implications of the association between polyphenol intake and sleep and to inform future research. The effects of polyphenol intake, including chlorogenic acid, resveratrol, rosmarinic acid, and catechins, on sleep quality and quantity are discussed to identify polyphenol molecules that may improve sleep. Although some animal studies have investigated the mechanisms underlying the effects of polyphenols on sleep, the paucity of trials, especially randomized controlled trials, does not allow for conducting a meta-analysis to reach clear conclusions about the relationships among these studies to support the sleep-improving effects of polyphenols.

## 1. Introduction

Sleep is essential for human body homeostasis, circadian rhythms, as well as metabolism, organ function, and other important physiologic functions, regulate the sleep-wake cycle [1]. The endogenous 24-h circadian rhythm is generated by the molecular clock, which is controlled by environmental factors. The circadian clock is the master regulator of physiologic functions, and disruption of the circadian rhythm severely affects health. The sleep-wake cycle is controlled by circadian rhythms that fluctuate on the basis of various factors, including diurnal variations in body temperature [2], blood pressure and pulse [3,4], and endocrine hormone secretion [5,6]. Sleep disturbances are well recognized to lead to psychologic distress, physical dysfunction, and reduced quality of life [7]. Sleep quality and quantity are critical factors in achieving a healthy quality of life, and inadequate sleep is a potential risk factor for cerebrovascular disease, depression, and mortality [8,9]. Sleep disturbances are a major social issue in the United States, Europe, and Asia [10,11,12]. Disturbances in circadian rhythms caused by abnormal sleep patterns, irregular work shifts, and airplane travel across time zones are associated with an enhanced risk of developing various diseases [13,14]. Although diet appears to influence the circadian clock, few studies, especially intervention studies in humans, have demonstrated a clear relationship between the intake of specific food components and circadian clock activities [15,16].

Extensive evidence suggests that fruit and vegetable intake affects body weight and risk of chronic diseases, which may be associated with sleep disturbances [17,18,19]. Polyphenols found in many plant foods also exhibit diverse physiologic functions and have protective effects against certain diseases, such as coronary artery disease [20,21,22]. Notably, only a few studies have investigated the relationship between polyphenols and improved sleep quality, duration, and onset time in obese and standard-weight individuals [23]. Godos et al. [24] performed an epidemiologic study to investigate the population demographic and dietary characteristics of 1936 adults inhabiting southern Italy, in order to determine the potential association of dietary polyphenol intake and sleep quality. Individuals with a high intake of flavonoids such as flavanones, flavones, phenolic acids (i.e., hydroxycinnamic acid), and lignans, had a significantly lower likelihood of inadequate sleep quality. These associations became even clearer when participants were stratified by weight; the effects were significant for normal-weight individuals but not for overweight/obese individuals. In addition, the UK Women’s Cohort Study followed 13,958 women for approximately 4 years to assess the possible association between intake of fruit- and vegetable-derived dietary polyphenols and duration of sleep [25]. Their findings revealed a direct association between total intake of fruits and vegetables and the estimated total polyphenol content and duration of sleep, but no association between subgroups of polyphenols and sleep duration was detected [25]. Many factors contribute to sleep, but nutrients are considered to be among the most important factors affecting various sleep parameters. Strategies that ensure the proper timing of meals are thought to produce positive sleep outcomes [26]. In addition, high-protein diets containing essential amino acids, low glycemic index foods, and antioxidant-rich fruits are reported to contribute to better sleep quality [27,28,29].

St-Onge et al. [30] demonstrated that a plant-based diet may reduce the risk of developing cardiovascular disease by improving sleep. Recent clinical trials revealed that consumption of kiwifruit [31] or tart cherry juice [32] improved sleep (sleep efficiency, sleep quality, and insomnia) in healthy adults, as well as older adults. The relationship between sleep and the intake of polyphenols abundant in fruits and vegetables, however, remains unclear. From a clinical perspective, only a few published literature reviews have investigated the effects of polyphenol intake on sleep. Polyphenols have antioxidant and anti-obesity effects and improve vascular endothelial function. Numerous studies have reported beneficial effects of polyphenol intake on the autonomic nervous system activity and the intestinal microbiota [33,34]. Clarifying the effects and mechanisms of action of polyphenols on sleep will contribute to the body of knowledge on sleep-related health. 

This review examines and discusses the effects of polyphenol intake on sleep, with particular emphasis on clinical and epidemiologic studies in humans and preclinical animal studies.

## 2. An Overview of Polyphenol Metabolism

Polyphenols are natural compounds with a structure containing at least two phenyl rings and at least one hydroxyl substituent. Polyphenols are found in plants, vegetables, grains, fruits, coffee, and tea. Polyphenols are secondary plant metabolites synthesized in plant tissues, and their structure provides protection against pathogens, ultraviolet irradiation, and oxidative injury [35,36]. Plant maturity at harvest, climatic conditions, infectious processes, and post-harvest processing and storage can affect both the polyphenol composition and content [37]. Polyphenols are bioactive phytochemicals comprising subclasses of flavonoids, phenolic acids, stilbenes, and lignans [38,39]. 

The flavonoid structure comprises a chroman ring attached to a second aromatic ring, and includes flavanols (e.g., quercetin in onions), flavanones (e.g., hesperidin in oranges), isoflavones (in soybeans), anthocyanins (in blueberries), and flavan-3-ols (e.g., epigallocatechin gallate in green tea) [40,41,42]. Moreover, several groups of simple phenols with diphenylpropane structures, such as lignans, stilbenoids, tannins, and phenylpropanoids, have been identified [43]. Flavonoids are glycoside and non-glycoside conjugates present in plants, and their bioavailability in humans depends on their moieties [38,44]. Dietary flavonoids are hydrolyzed enzymatically in the gastrointestinal tract, subsequently absorbed in the intestine, and then conjugated by phase II enzymes into the glucuronide/sulfate form in epithelial cells and the liver [45,46]. Although some flavonoids are absorbed in the small intestine, most are transported to the large intestine, where deconjugated metabolites and aglycons are further broken down by the colonic microflora for easy absorption of molecules such as phenolic acids [45]. For example, quercetin-3-O-rutinoside from tomatoes is absorbed primarily in the large intestine, where multiple methylation and glucuronidation products are present, and metabolized by the intestinal bacterial flora [44]. Green tea flavan-3-ols are primarily metabolized in the small intestine and then enter the colon, where they are further broken down by the microflora to produce phenolic acids [47]. Intestinal bacteria as well as phenolic and polyphenolic compounds are considered critical for the absorption of numerous flavonoids and influence their bioavailability in the systemic circulation. Moreover, polyphenols remain in the colon longer than in the small intestine and thus have a high potential to influence the colonic microbiota and colon health [48].

Dietary-derived polyphenols have antioxidant, anti-inflammatory, and antiallergic effects in humans. They reduce and prevent aging-related diseases and may also be helpful against cardiovascular events, cancer, osteoporosis, diabetes, and neurodegenerative diseases in humans [49,50,51]. In vitro studies suggest that the direct antioxidant effects of polyphenols underlie their health benefits. These antioxidant effects may not be relevant in the human body, however, because the concentrations of scavenging free radicals reached after oral ingestion are not sufficient to exert significant effects in most tissues [39,52,53,54]. Nevertheless, many other biochemical and molecular mechanisms via multiple intra- and intercellular signaling pathways may underlie their effects, such as regulation of nuclear transcription factors, fat metabolism, and synthesis of inflammatory mediators (e.g., cytokines) [55,56]. Flavonoids are involved in glucoregulation through downstream signals such as increasing insulin secretion, decreasing apoptosis, promoting beta cell proliferation, and reducing insulin resistance, inflammation, and oxidative stress in muscle and other cells. Phlorizin, an apple dihydrochalcone, specifically and competitively inhibits sodium-dependent glucose transporter 1 (SGLT-1) in the gut and SGLT-2 in the kidney and may be useful for treating hyperglycemia [57]. Several systematic studies have demonstrated the antioxidant, anti-inflammatory, and other complex biologic roles of polyphenolic compounds, particularly their protective effects against metabolic disorders and chronic diseases [58]. Polyphenols may also act as bioactive substances that enhance the body’s immune system and inhibit cellular inflammation and tumor angiogenesis [59,60]. While the potential of polyphenols to prevent disease may be due primarily to their antioxidant effects, they also induce targeted pharmacologic effects as well as epigenetic changes [33,61]. 

## 3. Sleep Assessment Methods

Sleep can be objectively assessed by polysomnography (PSG), electroencephalography (EEG), and actigraphy. PSG was first used in clinical settings in the 1970s and is widely regarded as the gold standard method for assessing sleep characteristics [62,63]. Typical PSG studies include central, frontal, and occipital EEG, recordings of eye and jaw movements, and other types of muscle activity (electromyography), an electrocardiogram, pulse oximetry, and monitoring of spontaneous breathing, nasal and oral airflow, and body position [64]. Various other sensors can also be integrated depending on the needs and circumstances of the individual study [65]. The data obtained by PSG are qualitatively and quantitatively accurate and can describe the sleep architecture, i.e., the sequence of the different sleep stages over the course of a night. The brain state during sleep consists of a cycle of two alternating phases: non-rapid eye movement sleep (slow wave sleep [SWS]) and rapid eye movement (REM) sleep. SWS is characterized by high-amplitude slow oscillations (<1 Hz) and sleep spindles (0.5–2-s bursts of 10–16 Hz), and REM sleep is characterized by low-amplitude, fast oscillating EEG activity (4–11 Hz) similar to that in the awake state [66,67,68]. Although PSG is the gold standard for sleep measurement, it is complicated, expensive, and time-consuming, and the equipment set-up and data output analysis require a high degree of expertise. Single-channel EEG signal meters were recently applied to research to reduce the effort required [69]. Koley et al. [70] utilized 39 time-domain, frequency-domain, and nonlinear features of EEG signals to develop a vector-based machine learning algorithm that automates sleep classification with an accuracy of 85.7%. Further advances in these areas of research are expected to lead to more accurate assessments in this field.

Actigraphy is a method of continuously monitoring the amount of body movement and activity to objectively estimate sleep and wake behavior. Actigraphic devices typically include a 3-axis or 2-axis accelerometer that records movement as a function of time based on an algorithm; sleep/wake behavior is calculated and the activity/inactivity cycles are converted to reflect wake/sleep, respectively. Thus, sleep is indirectly measured as the average of an individual’s movement activity. The wake/sleep data can be used to calculate several variables related to sleep duration and sleep quality, such as total sleep time, sleep onset latency, sleep efficiency (total sleep time/total time in bed), and wake after sleep onset.

Researchers may utilize sleep diaries and questionnaires to assess subjective estimates of sleep-related parameters. Sleep questionnaires are often used as a primary tool to screen for sleep disorders [71,72] on the basis of their characteristics. To assess overall sleep quality, standardized sleep diaries [71], the Pittsburgh Sleep Quality Index [73], the Athens Insomnia Scale [74], the Oguri-Shirakawa-Azumi sleep inventory MA version [75], the Berlin Questionnaire [76], and the Epworth Sleepiness Scale [77] are used. For example, the Pittsburgh Sleep Quality Index assesses 7 dimensions of sleep quality, latency, duration, habitual sleep efficiency, sleep disturbances, sleep medication, and daytime dysfunction over the previous month. Scores for each dimension are summed, providing a global sleep quality index, with higher scores indicating lower sleep quality [73]. When other sleep monitoring methods are difficult to use, a validated questionnaire can provide valuable information and fill gaps in existing evidence. Self-administered diaries are used to collect a variety of sleep-related data, such as bedtime/wake time, lights-out time, naps, daytime sleepiness, alertness, caffeine and alcohol intake, and exercise habits [71]. For a meaningful assessment, participants should monitor and record their sleep characteristics in the diary for at least a week, but this may diminish compliance. 

## 4. Studies of the Effects of Polyphenols on Sleep

Caffeine is a xanthine alkaloid often included as a component of polyphenol-rich beverages such as coffee and tea. Clinical and pharmacologic evidence indicates that caffeine impairs sleep [78,79,80]. Therefore, the caffeine status should be noted in clinical sleep studies. 

### 4.1. Chlorogenic Acids

Chlorogenic acids (CGAs) are phenolic compounds widely found in plant seeds and/or bodies, such as coffee beans, sweet potatoes, potatoes, apples, and burdock roots. CGAs are mainly known for their antioxidant properties [81] and vascular endothelial function-improving effects [82,83,84] and are reported to improve hypertension [85]. In addition, CGAs have a wide range of other beneficial effects, such as reducing body fat [86], improving cognitive function [87,88,89], and improving skin conditions [90]. Recent studies examined the possible effects of continuous consumption of CGA in the form of a coffee bean extract from which the caffeine was removed on human sleep and the autonomic nervous system. Table 1 summarizes the clinical trials that examined the effects of CGA on sleep. Ochiai et al. [91], who studied healthy adult males who consumed a beverage containing 300 mg of decaffeinated CGA derived from green coffee bean extract for 2 weeks, reported that the CGA group experienced less fatigue upon waking and significantly improved subjective sleep quality. Park et al. [92], in a study of nine healthy men and women who consumed a test beverage containing 600 mg of decaffeinated CGA or a placebo for 5 days, reported that CGA decreased sleep latency as measured by sleep EEG and increased parasympathetic activity as determined by measuring heart rate variability. In a recent randomized controlled trial (RCT) of healthy older adults, Saitou et al. [87] found that consumption of 300 mg of coffee bean extract for 16 weeks may improve cognitive function (motor speed, psychomotor speed, and executive function). These findings are interesting, given the reported association between improved sleep and improved cognitive function. 

Shinomiya et al. [94] studied how CGA and caffeic acid at doses of 500 and 200 mg/kg, respectively, affected the sleep-wake cycle in rats. They observed no significant effects of CGA and its metabolites on any sleep state and therefore could not investigate the mechanism of the effects of CGA on sleep architecture or sleep quality. While CGA is reported to have potential antidepressant effects, animal behavior studies with CGA revealed neuronal protective effects in the brain and promotion of serotonin release [95,96]. Studies of growing pigs showed that CGA supplementation increased the diversity of the gut microbiota and thus significantly augmented aspartic acid, threonine, alanine, and arginine in the serum, as well as serotonin (5-HT, 5-hydroxytryptamine) levels in the large intestine [97]. Low serotonin levels are associated with insomnia and sleep rhythm disorders, as well as depression [98]. 

Ferulic acid (4-hydroxy-3-methoxycinnamic acid), a typical bioactive metabolite of CGA, is a potential mediator of shortened sleep latency. Tu et al. [99] reported that ferulic acid significantly potentiated pentobarbital-induced (45 mg/kg, i.p.) sleep by prolonging the sleep time and shortening the sleep latency in mice, in a dose-dependent manner. One possible mechanism by which CGA intake improves sleep structure and quality may be its effects on autonomic nervous system activity. Werner et al. [100] demonstrated that higher resting high-frequency heart rate variability was associated with higher subjective (i.e., Pittsburgh Sleep Quality Index) and objective (i.e., PSG) sleep quality in 29 healthy young women during an extended neutral film clip. Park et al. [92] also demonstrated that CGA increases parasympathetic activation and reduces sleep latency. Furthermore, acute psychologic stress decreases sleep quality and reduces parasympathetic activity during sleep [101]. These findings suggest that CGA inhibits the effects of psychologic stress and other sleep quality-impairing factors via the autonomic nervous system, which promotes recovery from sleep-induced fatigue [91].

### 4.2. Resveratrol

Resveratrol, classified as a stilbenoid, is abundant in grape seed skins, mulberries, pea pod skins, wine, and tea. Resveratrol appears naturally as a cis and trans compound and has been identified as an activator of sirtuin 1 (SIRT1) [102]. SIRT1 is an NAD^+^-dependent histone deacetylase family member that regulates life span by its protective effects against metabolic stress, such as obesity caused by a high-fat diet [103]. Compounds such as resveratrol that activate SIRT1 are expected to prevent the onset of metabolic disorders and promote healthy aging. Animal studies showed that resveratrol enhances metabolic health by increasing insulin sensitivity and mitochondrial function in skeletal muscle [104]. Conflicting findings, however, are reported by human clinical trials. While some studies report significant improvements in metabolic health-related parameters [105,106,107], other studies report no effect [108,109,110,111]. A recent systematic review and meta-analysis of 17 RCTs involving 871 patients with type 2 diabetes mellitus revealed a superiority of resveratrol compared with placebo for improving fasting blood glucose and other parameters [112]. Resveratrol exhibits a variety of beneficial effects in neurodegenerative and neurocognitive disorders [113] and is also expected to have beneficial effects on sleep.

Only a few clinical trials have evaluated the effects of resveratrol on sleep, as shown in Table 2. In a study examining the effect of resveratrol intake for 6 months on insulin sensitivity, no differences in sleep and life qualities were detected between the resveratrol and placebo treatment groups, as assessed by a questionnaire that included a secondary endpoint [111]. In a questionnaire-based study by Pennisi et al. [114] to evaluate the effects of resveratrol on both sleep quality and sleep disturbances in hepatitis C patients receiving antiviral treatment with interferon, resveratrol supplementation improved sleep quality and quantity, and decreased sleep disturbances. Wightman et al. [115] reported that chronic supplementation with 500 mg/day of trans-resveratrol in healthy subjects improved cognitive function, but no significant treatment-related differences in sleep quality were detected by the Pittsburgh Sleep Quality Index and its subcomponents compared with placebo. Resveratrol supplementation did, however, reduce symptoms of associated anxiety, depression, and sleep disturbances, suggesting its potential anxiolytic effects. 

Pifferi et al. [116] investigated the effects of resveratrol administration on the EEG rhythm-based sleep-wake cycle in a non-human primate, the grey mouse lemur (Microcebus murinus). After three weeks of resveratrol administration, the percentage of active wake time was significantly increased, with the increase occurring mostly during the resting phase of the sleep-wake cycle. The increase in active wake time caused by resveratrol- came at the expense of paradoxical sleep and SWS, which were significantly decreased. Dietary resveratrol may be involved in regulating the circadian clock in experimental animals [117]. Energy balance in several species is modulated by resveratrol activating several proteins that are part of the energy-regulating pathways, such as PGC1α [118] and SIRT1 [119]. SIRT1 is involved in biologic clock processes [120,121], and regulation of this protein by resveratrol may cause changes in the biologic rhythm patterns of physiologic parameters. Recent studies in young animals showed that dietary supplementation with resveratrol can alter the structure of sleep-wake rhythms by reducing the amount of SWS and increasing activity [116]. How resveratrol affects sleep, especially in humans, remains unclear, and the mechanisms are mostly unknown.

### 4.3. Rosmarinic Acids

Rosmarinic acid is a natural polyphenol in spearmint (*Mentha spicata*), shiso (*Perilla frutescens*), rosemary (*Rosmarinus officinalis*), lemon balm (*Melissa officinalis*), and other plants [122,123]. Rosmarinic acid has antioxidant, antibacterial, antiviral, anti-inflammatory, analgesic, neuroprotective, and cardioprotective effects against both gram-positive and gram-negative bacteria [124]. Rosmarinic acid may also support cognitive function in relation to a common polyphenolic component. This naturally occurring phenolic acid is the esterification product of caffeic acid and 3,4-dihydroxyphenyl lactic acid. In Table 3, a randomized, double-blind, placebo-controlled, parallel study was performed to assess cognitive function, sleep, mood, and quality of life by a validated questionnaire in healthy men and women (*n* = 142) taking 900 mg per day of a proprietary spearmint (*Mentha spicata*) extract (≥14.5% rosmarinic acid and 24% phenolic content) or placebo for 90 days [125]. The results showed that the aqueous extract improved cognitive function but did not significantly affect mood, sleep, or quality of life.

The in vitro inhibition of gamma-aminobutyric acid (GABA) transaminase by rosmarinic acid suggests that the activation of GABA_A_-ergic system is a potential treatment for insomnia [126]. In a pentylene tetrazole-induced kindling mouse model, 4 mg/kg of rosmarinic acid bound to diazepam shortened sleep latency in the diazepam-induced sleep time test [127]. In a recent study, the sleep-promoting effects of rosmarinic acid, which targets the adenosine receptor (a therapeutic target for insomnia), were evaluated by pentobarbital-induced sleep studies in mice, EEG and electromyography, and immunohistochemical techniques [128]. Simultaneously, the underlying mechanisms were evaluated by pharmacologic approaches using antagonists of the adenosine A1 receptor and the adenosine A2a receptor (8-cyclopentyl-1,3-dipropylxanthine and SCH5826, respectively). The results demonstrated that rosmarinic acid has direct binding activity and agonist activity against adenosine A1 receptors. Rosmarinic acid decreased sleep latency and increased total sleep time in a mouse pentobarbital (42 mg/kg, i.p.)-induced sleep model [129]. Rosmarinic acid also increased the effects of sub-hypnotic pentobarbital (28 mg/kg, i.p.) on sleep time and the number of mice that fell asleep [129]. EEG recordings in rats showed that rosmarinic acid (2.0 mg/kg) not only reduced the number of sleep/wake cycles and REM sleep but also enhanced total and non-REM sleep [129].

**Table 3:** Summary of human clinical studies assessing the association between sleep and RA consumption.

Author (Year)	Study Design	Population	Primary Outcome	Sleep Assessment	Sample (*n*)	Duration	Intervention (Caffeine)	Control	Results on Sleep	Reference
Tubbs et al. (2021)	RCT, parallel	Healthy men and women	Sleep, daytime functioning	Activity tracker, Sleep diary, ISI, PSQI	105	30 days	120 mg/d RA and EGCg < 4.85 mg caffeine	Placebo	RA and EGCg improved daily sleep quality (*p* = 0.008) and reduced insomnia severity (*p* = 0.044)	[130]
Falcone et al. (2019)	RCT, parallel	Healthy men and women	Cognitive performance, sleep	Questionnaire (LSEQ, PSQI)	142	90 days	130 mg/d RA	Placebo	No differences in the quality of seep were detected	[125]
Herrlinger et al. (2018)	RCT, parallel	Healthy men and women	Cognitive performance	Questionnaire (LSEQ)	90	90 days	900 mg/d or 600 mg/d spearmint extract	Placebo	Spearmint extract improved the ability to fall asleep (*p* = 0.0046).	[131]

Abbreviations: EGCg, epigallocatechin gallate; ISI, insomnia severity index; LSEQ, Leeds sleep evaluation questionnaire, RA, rosmarinic acid; RCT, randomized controlled trial; PSQI, Pittsburgh Sleep Quality Index.

### 4.4. Catechins

Catechins (flavanols) are the predominant polyphenols in tea. Green tea is rich in catechins (e.g., epicatechin, epicatechin gallate, epigallocatechin, and epigallocatechin gallate [EGCg]) and their thermal isomers (e.g., catechin, catechin gallate, gallocatechin, and gallocatechin gallate) [132,133]. Many of the physiologic functions of catechins (e.g., antioxidant, antidiabetic, and antiatherosclerotic effects) have been established in vitro, in animal studies, and in human trials [134,135,136,137,138].

Unno et al. [139] examined the effects of a low-caffeine green tea (caffeine content was reduced to 20–25%) on the stress responses and sleep parameters in 20 middle-aged men and women; the results were compared with those of standard catechin and theanine-containing teas (Table 4). Sleep parameters were measured with a single-channel EEG, the stress response was measured as salivary α-amylase activity, and subjective fatigue was assessed with a questionnaire. Compared with standard caffeine-containing tea, low-caffeine green tea consumption improved fatigue and tiredness but had no significant effect on sleep. Zhang et al. [140] conducted a crossover study of three groups of 12 non-obese men who consumed oolong tea (100 mg caffeine, 21.4 mg gallic acid, 97 mg catechins, and 125 mg polymerized polyphenols), caffeine (100 mg), or a placebo over a 14-day period. On day 14 of each session, energy metabolism by indirect calorimetry and sleep by PSG were measured. Caffeine and oolong tea consumption increased fat oxidation, and caffeine consumption increased parasympathetic nervous activity but had no effect on sleep parameters.

A recent review discussed the effects of tea components (L-theanine, caffeine, tea polyphenols [catechins], tea pigments, tea polysaccharides, and GABA) on sleep and brain function [141]. After ingestion of green tea, catechins and their main component EGCg can easily transit the blood-brain barrier and distribute in the brain, exerting neuroprotective effects; therefore, EGCg may regulate sleep [142,143]. EGCg binds to GABA_A_ receptors in vitro [144], and the relative levels of EGCg and epigallocatechin in low-caffeine green tea may modulate the effects of GABA through binding to GABA_A_ receptors [145]. Furthermore, catechins suppressed the expression of tumor necrosis factor α and reduced cognitive deficits in a mouse model of sleep deprivation [146], and EGCg, like the GABA_A_ receptor agonist muscimol, prolonged the duration of pentobarbital-induced sleep and reduced sleep latency in mice [147]. Vocalizations that normally occur during social isolation stress were suppressed when EGCg (50, 100, and 200 μg) was administered intracerebroventricularly to chickens under acute stress conditions, and a dose-dependent reduction in wakefulness time and induction of sleep-like behavior were observed [148]. Although these basic studies in animals demonstrate the potential sleep-improving effects of catechins and EGCg without caffeine, further clinical investigation, especially in humans, is required. 

## 5. Discussion

The present review examines the effects of polyphenol intake on sleep, with particular emphasis on human clinical and epidemiologic studies and preclinical animal studies. The relationship between polyphenol intake, which has many favorable health aspects, and sleep improvements has not been adequately investigated and remains unclear. Few reviews have examined the effects of polyphenols specifically and their intake on sleep, especially from a clinical perspective. Polyphenols exhibit many beneficial effects, including antioxidant and antiobesity effects, as well as effects on vascular endothelial function, and many studies have evaluated their effects on the autonomic nervous system and gut microbiota. Clarifying the effects of polyphenol intake on sleep would contribute to this body of research and guide future research. To our knowledge, this review is one of very few literature surveys to identify polyphenol molecules that may improve sleep quality. Due to the limited number of studies evaluating the effects of polyphenols on sleep, we could not conduct a meta-analysis to draw definitive conclusions about the relationships among the studies. Further, a dose-response meta-analysis integrating the studies was also not available, and thus the dose responsiveness could not be considered. 

This review examines the effects of the dietary polyphenol intake (i.e., CGA, resveratrol, rosmarinic acid, catechins, and lignans (phenylpropanoids)) on sleep. In addition, we discussed the absorption and metabolic properties of polyphenols and subjective, as well as objective, assessment methods of sleep. Sleep, especially when it is self-reported in questionnaires, cannot be completely excluded from biases (e.g., placebo effects). Therefore, studies in which sleep EEG is measured by appropriate methods and sleep state is determined are essential to determining the true effects of the intake of polyphenol components on sleep. A review of the literature revealed that only a few human studies have sufficiently investigated the pharmacologic mechanisms of polyphenols, and the types of polyphenols that can be concluded to be effective in improving sleep are limited. A small human study [92] demonstrated the potential efficacy of CGA for improving sleep on the basis of objective sleep measurements by EEG and discussed the mechanisms by which it may improve sleep, such as through ferulic acid, a CGA metabolite. No meta-analysis of CGA has been conducted, however, and further research, especially in human studies with large sample sizes, is warranted. 

Several different neuronal systems regulate the sleep-wake cycle, including GABAergic, serotonergic, histaminergic, and adrenergic systems. The GABAergic system is a major sleep-promoting pathway with an important role in actions of many hypnotic drugs. Serotonergic activity is responsible for not only sleep, but also sleep inhibition and arousal. Serotonin has complex effects on the sleep-wake cycle, which may be due to its actions in different regions of the brain involved in sleep and wakefulness [149]. Ferulic acid, a phenolic acid categorized as a hydroxycinnamic acid, is a typical metabolite of CGA and may partially mediate sleep improvement. Ferulic acid binds to receptors in the GABAergic and serotonergic neurotransmitter systems and is suggested to affect sleep. Ferulic acid is found in many plants, including cabbage, wheat, rice bran, tomatoes, and onions, but its pharmacokinetic properties, such as low gastrointestinal stability, a short plasma half-life after oral administration, and low bioavailability limit its use in the treatment of sleep. More than 70% of ferulic acid is absorbed from the gastrointestinal tract within 30 min after oral administration and is rapidly excreted by the kidneys, resulting in a short residence time and low plasma concentrations. Furthermore, despite its ability to cross the blood-brain barrier, ferulic acid concentrations in the brain are very low, limiting its use in the treatment of sleep [150,151]. Therefore, ferulic acid as a metabolite of polyphenols such as CGA may beneficially affect sleep due to its improved bioavailability, stability in the gastrointestinal tract, and availability in the blood. Tu et al. [99] evaluated the hypnotic and sedative effects of ferulic acid and found that ferulic acid exerts sedative effects by suppressing motor activity; ferulic acid also significantly potentiates the hypnotic effects of pentobarbital, shortens sleep latency, prolongs sleep duration, and increases the rate of sleep onset. Rosmarinic acid inhibits GABA transaminases, suggesting an effect on sleep through activation of the GABAergic system [126]. Furthermore, rosmarinic acid enhances pentobarbital-induced sleep behavior via GABA_A_ neurotransmission, suggesting that the effects of rosmarinic acid on sleep are mediated through the GABA_A_-ergic system [129]. Interestingly, CGA supplementation increases the diversity of the gut microbiota, and through these changes, significantly enhances serum aspartate, threonine, alanine, arginine, and colonic serotonin (5-HT) levels, which may lead to improvements in insomnia and sleep rhythm disorders [97]. Polyphenols are known to affect tryptophan metabolism through the gut microbiota [152]. Some effects seem inconsistent, however, which is attributed to changes in the bioavailability of polyphenols depending on the composition of the microbiota. The effects of polyphenols and their metabolites on sleep and sedative effects may be mediated by neural effects of the GABAergic or serotonergic neurotransmitter systems. 

In a systematic review and meta-analysis of supplements that may improve sleep, Chan et al. [153] found that adding amino acids, melatonin, and vitamin D to the diet improved subjective sleep quality, but, because of insufficient studies, especially RCTs, it is unclear whether or not the addition of zinc, resveratrol, magnesium, and nitrates to the diet also improves sleep quality. The authors considered that more RCTs should be conducted to examine the effects of resveratrol, a polyphenol, in various populations. In general mechanistic terms, metabolites of polyphenols can traverse the blood-brain barrier to varying degrees depending on the lipophilicity, while the less polar polyphenol metabolites can be taken up by the brain [154]. In the central nervous system, the major potential beneficial effects of dietary polyphenols include inhibiting neuronal apoptosis, modulating signaling pathways involved in neuronal survival, and stimulating neurogenesis [155,156]. With respect to specific mechanisms related to sleep characteristics, dietary polyphenols enhance recovery from sleep deprivation [157]. Furthermore, derivatives of hydroxycinnamic acid, such as ferulic acid, are GABA receptor agonists that act synergistically with 5-hydroxytryptophan, which has a sedative effect on motor activity, prolongs sleep duration, reduces sleep latency, and is involved in sleep quality [99,158]. The synergistic effects between polyphenols and other nutrients with distinct pharmacologic functions, such as phytosterols, should also be considered in future studies [159]. Lignans protect the blood-brain barrier from inflammatory cells by their antioxidant and anti-inflammatory properties in neurons, reducing oxidative stress, inflammation, and permeability [160,161,162]. Dietary polyphenols may improve endothelial dysfunction and help control blood pressure [163,164], and the improved endothelial function, along with decreased rates of REM sleep and increased REM sleep latency, may be associated with improved sleep quality [165,166]. The gut microbiota also influences the brain and behavior associated with anxiety and depression symptoms in response to modulation through dietary polyphenol intake [167]. Recent studies suggested that dietary polyphenols play a role in modulating the metabolism of the gut microbiota and that fluctuations in the gut microbiota can affect activity through polyphenol metabolites [168]. The current evidence, however, is based primarily on cellular and animal studies, and human studies are needed to identify specific metabolic types associated with activity in the brain.

This literature review was performed to identify polyphenol molecules with the potential to improve sleep quality and to reveal the potential role of individual polyphenols on sleep. The findings presented in this review should be considered in light of several limitations. First, this study was not a comprehensive review of the literature, which may lead to potential oversights (in particular, studies in which the effects on sleep are not the primary endpoints). It is also possible that some relevant studies published in non-English languages were missed. Despite the fact that herbs are very popular in Asia, no Chinese or Japanese language studies were included. Improving the quality of clinical studies published in those local languages could be improved, which would allow them to be included in future meta-analyses. Second, the discussion of mechanisms was drawn only from the studies and reports cited in the current review. Third, sleep quality and quantity were not considered, even though they may be related to some mental health issues.

## 6. Conclusions

A literature review was conducted to identify polyphenol molecules that may improve sleep quality. Several animal studies have examined the mechanistic effects of CGA, resveratrol, rosmarinic acid, and catechins on sleep, but the limited number of studies, especially RCTs, evaluating the effects of polyphenols on sleep did not allow for a meta-analysis to reach clear conclusions about the relationships among these studies. The dose responsiveness could not be considered because of the lack of a dose-response meta-analysis merging the studies.

## Figures and Tables

**Table 1:** Summary of human clinical studies assessing the association between sleep and chlorogenic acid consumption.

Author (Year)	Study Design	Population	Primary Outcome	Sleep Assessment	Sample (*n*)	Duration	Intervention (Caffeine Content)	Control	Results on Sleep	Reference
Ochiai et al. (2018)	RCT, crossover	Healthy men aged 30–54 y	Fatigue and sleep	Activity meter, questionnaire (VAS)	16	2 weeks	300 mg/day of CGA < 3 mg caffeine/100 mL	Placebo	CGA significantly improved sleep quality assessed by VAS (*p* < 0.05) and sleep efficiency assessed by an activity meter compared with the control (*p* = 0.046)	[93]
Park et al. (2017)	RCT, crossover	Healthy young men and women	Energy metabolism and sleep	PSG	9	5 days	without caffeine	Placebo	CGA significantly shortened sleep latency compared with the control (*p* = 0.043)	[92]

Abbreviations: CGA, chlorogenic acid; PSG, polysomnography; RCT, randomized controlled trial; VAS, visual analogue scale.

**Table 2:** Summary of human clinical studies assessing the association between sleep and resveratrol consumption.

Author (Year)	Study Design	Population	Primary Outcome	Sleep Assessment	Sample (*n*)	Duration	Intervention	Control	Results on Sleep	Reference
Ligt et al. (2020)	RCT, parallel	Overweight men and women	Insulin sensitivity	Questionnaire (PSQI)	41	6 months	150 mg/d of trans- resveratrol	Placebo	No differences in sleep quality were detected	[111]
Pennisi et al. (2017)	RCT, parallel	Hepatitis C patients	Quality of sleep	Questionnaire (PSQI, ESS)	60	12 months	19.8 mg/d resveratrol	Placebo	Resveratrol significantly improved sleep quality	[114]
Wightman et al. (2015)	RCT, parallel	Healthy men and women	Cognitive performance	Questionnaire (PSQI)	60	4 weeks	500 mg/d of trans- resveratrol	Placebo	No differences in sleep quality were detected	[115]

Abbreviations: ESS, Epworth sleepiness scale; RCT, randomized controlled trial; PSQI, Pittsburgh Sleep Quality Index.

**Table 4:** Summary of human clinical studies assessing the association between sleep and catechin consumption.

Author (Year)	Study Design	Population	Primary Outcome	Sleep Assessment	Sample (*n*)	Duration	Intervention (Caffeine)	Control	Results on Sleep	Reference
Zhang (2020)	RCT, crossover	Healthy men aged 20–56 y	Energy metabolism	PSG, Questionnaire (PSQI)	12	2 weeks	48.5 mg/d catechins, 51.8 mg/d caffeine	Placebo, 51.8 mg caffeine	No differences in the sleep parameters were detected	[140]
Unno et al. (2017)	RCT, crossover	Healthy men and women	Stress responses, sleep parameters	Single-channel EEG	20	1 week	Standard green tea	Low- caffeine green tea	No significant difference in sleep parameters was detected	[139]

Abbreviations: EEG, electroencephalogram; ISI, insomnia severity index; LSEQ, Leeds sleep evaluation questionnaire, RCT, randomized controlled trial; PSQI, Pittsburgh Sleep Quality Index.

## Data Availability

Not applicable.
